# Diagnostic accuracy of cardiac MRI, FDG-PET, and myocardial biopsy for the diagnosis of cardiac sarcoidosis: a protocol for a systematic review and meta-analysis

**DOI:** 10.1186/s41512-020-00073-4

**Published:** 2020-05-07

**Authors:** Dominik Roth, Nikolaos Kadoglou, Mariska Leeflang, Rene Spijker, Harald Herkner, Marialena Trivella

**Affiliations:** 1 Centre for Statistics in Medicine, NDORMS, University of Oxford, Windmill Road, Oxford, OX 3 7LD UK; 2 Department of Emergency Medicine, Medical University of Vienna, Spitalgasse 23, 1090 Vienna, Austria; 3 Amsterdam UMC, University of Amsterdam, Meibergdreef 9, Amsterdam, The Netherlands

**Keywords:** Sarcoidosis, Cardiac, Diagnostic test accuracy, Systematic review (diagnostic), Indirect comparison, Bayesian meta-analysis

## Abstract

**Background:**

CS constitutes a rare but potentially underdiagnosed and fatal disease. Its diagnosis remains difficult owing to the infrequent and indistinguishable symptoms and the lack of formal diagnostic criteria dependent upon the diagnostic techniques used. Early diagnosis and treatment, however, may help to counter its poor prognosis.

We aim to characterize and compare the diagnostic accuracy of cardiac MRI, FDG-PET and myocardial biopsy for the diagnosis of cardiac sarcoidosis and to advance and compare methods for complex diagnostic test accuracy reviews and meta-analysis.

**Methods:**

Following a systematic review on DTA studies on the aforementioned topic, a four-part approach to meta-analysis will be used: (1) direct comparison of index tests with clinical reference standard, (2) indirect comparison of index tests with clinical reference standard, (3) addition of an alternative test to that indirect comparison (4) and Bayesian meta-analysis using results of part 3 as informative prior for comparisons analogous to part 1 and 2.

**Discussion:**

The most widely recognized diagnostic algorithm for cardiac sarcoidosis is considered out of date, as it precedes the introduction of imaging techniques in diagnostic pathways. These novel imaging techniques, like CMR and FDG-PET scan, have emerged as promising diagnostic tools which may fill the current diagnostic gap. Thus, a systematic review and evaluation of CS diagnosis are much needed. Such an attempt is anticipated to alter the current diagnostic guidelines for CS by shedding more light on the role of sophisticated imaging techniques on prompt CS therapy and follow-up.

**Trial registration:**

PROSPERO, CRD42019047126

## Background

### Target condition being diagnosed

Sarcoidosis is a multi-system inflammatory disorder of unknown aetiology resulting in the formation of non-caseating granulomas. The precise prevalence of sarcoidosis is largely unknown due to under-diagnosis, but it is thought to be geographically or ethically dependent and ranging between 4 and 40 per 100,000 [1]. Although the cause of this uncommon disease remains unknown, the onset is hypothesized to be precipitated by exposure to an unknown antigen with subsequent exaggerated immune response leading to granuloma formation in multiple organs [2].

In sarcoidosis patients, any organ may be affected, e.g. the lungs, skin, lymph nodes, eyes, heart and central nervous system. In the context of systemic disease, cardiac sarcoidosis (CS) refers to cardiac involvement of sarcoidosis. There are no specific symptoms for CS or they may be vague, mimicking other more frequent cardiovascular abnormalities (e.g., syncope, chest pain, dyspnoea). Cardiac signs of CS may illustrate a variety of pathologic manifestations as well. The most well-known are arrhythmias and sudden cardiac death. The latter is the leading cause of death (up to 85%) in patients with CS [3–5]. The underlying causes of sudden death are atrioventricular block, severe ventricular arrhythmia or asystolic arrest [6–8]. Besides this, myocardial infiltration with granulomatous and/or fibrosis may lead to signs of ventricular dysfunction [9]. Cardiomyopathy represents the second group of causes of death (25%). Less frequently, CS patients may present pericardial effusion or features of cardiomyopathies. The early recognition of CS development and related interventions (e.g. devices implantation) may prevent the above fatal consequences. Moreover, the favourable course of CS when treated with corticosteroids is once again a good argument to chase up early diagnosis and prompt treatment [10].

To date, CS diagnosis is challenging, since asymptomatic cardiac involvement in patients with pulmonary sarcoidosis is relatively frequent. In asymptomatic patients, CS may be present even in the absence of any abnormality on standard cardiac testing (e.g. ECG, echocardiography), may manifest as an abnormal electrocardiogram alone or may be recognized as an abnormality on sophisticated imaging techniques. On the other hand, the presence of non-specific symptoms may mislead to other diagnoses and loss of valuable time.

In summary, CS constitutes a rare but potentially underdiagnosed and fatal disease. Its diagnosis remains difficult owing to the infrequent and indistinguishable symptoms and the lack of formal diagnostic criteria dependent upon the diagnostic techniques used. Early diagnosis and treatment, however, may help to counter its poor prognosis [11].

### Reference standard

Timely diagnosis and treatment of cardiac involvement of sarcoidosis are vital, as outlined above. There are two mainstays of working-up diagnosis of CS: (1) histological diagnosis and (2) a cluster of clinical investigations. Nowadays, a conclusive diagnosis of CS is usually based on Japanese guidelines, while the ‘gold standard’ of CS diagnosis remains the demonstration of non-caseating granulomas on endomyocardial biopsy [12]. The latter is, however, an invasive technique with pretty low sensitivity, based on post-mortem analyses, which is additionally unsuitable for repeated use and follow-up monitoring. Reports suggest the sensitivity of detecting sarcoid granuloma on endomyocardial biopsy is around 20–30% [13]. This is entirely explained by the patchy involvement of disease, and despite the evolution in imaging techniques, guiding histological sampling has not improved its sensitivity [14]. A positive endomyocardial biopsy can establish a definitive CS diagnosis, but crucially, a negative biopsy does not exclude the disease. In order to overcome the disadvantages of histological diagnosis, the Japanese Society of Sarcoidosis and Other Granulomatous Disorders has proposed since 2006 a more clinical approach combining a cluster of laboratory investigations (ECG, echocardiography, nuclear perfusion, MRI, cardiac biopsy) for the diagnosis and follow-up of patients with suspected CS [15]. In this case, established diagnosis of extra-cardiac sarcoidosis is a prerequisite. This diagnostic algorithm of CS is internationally advocated; however, the proposed major and minor criteria used have received great criticism. For instance, there are no specific definitions of the mentioned abnormalities (e.g. no quantification of basal thinning of the ventricular septum or advanced atrioventricular block). Gallium cardiac uptake is not used anymore in clinical practice, while the emerging use of 18F-fluoro-2-deoxy-d-glucose (18-FDG) PET scan is not mentioned at all [16].

That blurred vision of CS diagnosis may explain the wide variation in the prevalence of CS in patients with established sarcoidosis along studies (from 3.7 to 54.9%) [17]. More recently, two sophisticated imaging techniques have emerged as alternatives for CS diagnosis; the cardiac magnetic resonance (CMR) and the 18-FDG PET scan. A finding from the former technique has already been adopted in Japanese guidelines, as a minor criterion. Both techniques are gaining ground rapidly on becoming the new ‘gold standard’.

For the purpose of this review, the Japanese criteria, as published from 2006 onwards, will serve as a reference standard (Table 1). Methods to deal with different versions of this standard, as well as the fact that some of the index tests might be included in the reference standard, are outlined in the “Methods” section.

**Table 1:** ‘Japan Criteria’ for the diagnosis of cardiac sarcoidosis [15]

**Histologic diagnosis group**	
Endomyocardial biopsy demonstrates epithelioid granuloma without caseating granulomatoma	
**Clinical diagnosis group**	
In patients with a histologic diagnosis of extra-cardiac sarcoidosis, cardiac sarcoidosis is suspected when ‘a’ and at least one of criteria ‘b’ to ‘e’ is present and other aetiologies such as hypertension and coronary artery disease have been excluded	
a) Complete right bundle branch block, left-axis deviation, atrioventricular block, ventricular tachycardia, premature ventricular contraction or pathological Q or S-T change on resting or ambulatory electrocardiogram	
b) Abnormal wall motion, reginal wall thinning or dilation of the left ventricle	
c) Perfusion defect by 201-thallium myocardial scintigraphy or abnormal accumulation by 67Ga-citrate or 99mTc-PYP myocardial scintigraphy	
d) Abnormal intracardiac pressure, low cardiac output, or abnormal wall motion or depressed ejection fraction of the left ventricle	
e) Interstitial fibrosis or cellular infiltration over moderate grade, even if the findings are non-specific	

### Index tests

CMR has shown a sensitivity of 75% and a good specificity (78%) [18, 19]. Findings consistent to CS include intra-myocardial focal zones with thickening, and increased signal intensity on both T2-weight and early gadolinium-enhanced images (oedema and inflammation). Regional wall motion abnormalities may be detected in cine CMR. Moreover, late gadolinium enhancement (LGE—related to collagenous scar) of the myocardium has been detected in granulomatous hearts. In CS, LGE is found predominantly in the mid-myocardium and epicardial areas, but rarely in the endocardium [20]. Notably, LGE is the only CMR finding included in the diagnostic algorithm of CS, as a minor criterion, although it is highly predictive of ventricular arrhythmias and poor outcomes [21, 22].

FDG-PET scanning is widely used in the evaluation of tumours, vasculitis and inflammatory diseases. Its use for CS diagnosis is characterized by high sensitivity (80–100%), but lower specificity (< 70%) [23]. In particular, the FDG-PET scan is more sensitive than CMR in detecting active sarcoid inflammation in the absence of myocardial oedema or scarring which might not be apparent on CMR. However, focal lesions are non-specific and not very well-localized. Moreover, the FDG-PET scan may differentiate CS from other non-inflammatory cardiomyopathies like ARVC [24]. Good preparation of patients may optimize the results of PET scanning [25].

Preliminary data implicate the superior sensitivity of FDG-PET scan over CMR in patients with documented pulmonary sarcoidosis and no previously diagnosed CS [26, 27]. However, a comparative evaluation of those two imaging techniques has not been done systematically. Scarce data derived from study cohorts with established or suspected CS. On the other hand, accumulating work has emphasized that CMR and PET scans will be complementary to one another for the investigation and follow-up of CS [28, 29].

A recent joint procedural position statement by the European Association of Nuclear Medicine, the European Association of Cardiovascular Imaging and the American Society of Nuclear Cardiology aims to standardize imaging for cardiac sarcoidosis [30].

### Clinical pathway

Patients with known or suspected systemic sarcoidosis (extra-cardiac) who develop cardiac symptoms and/or signs will be suspected of CS and should be intensively investigated for CS. Patients with sarcoidosis, who do not develop cardiac signs and/or symptoms, are still candidates of CS, and it is medically wise to be tested for cardiac involvement. Finally, CS belongs to a long list of cardiac diseases causing brady-arrhythmias or tachy-arrhythmias. In this case, CS may be part of the differential diagnosis; however, it is well down the line of investigations and usually appears as a last resort, after the exclusion of other common conditions. Thus, CS very rarely appears as a potential diagnosis in a patient presenting arrhythmia-related symptoms.

All patients with known or suspected systemic sarcoidosis should be referred for initial cardiologic examination. Diagnosis commonly follows a multi-step approach and could be rather variable and challenging. Patients might enter diagnostic workup either due to known extra-cardiac sarcoidosis, without any cardiac symptoms, or due to a clinical event of possible cardiac origin (e.g. syncope, arrhythmia), without previous suspicion of CS. One possible clinical pathway might be as follows:

Step 1: The first step in the diagnosis is the obtaining of a detailed medical/cardiac history, a thorough physical examination, an ECG and if it is normal an ambulatory ECG monitoring (the duration depends on the frequency of symptoms). The first step is always completed with transthoracic echocardiogram.

Step 2: If there are symptoms or any of the above tests in step 1 is abnormal, the patient will be investigated further. Regarding the subtle presence of CS in many patients, the existence of cardiac symptoms in patients with systemic sarcoidosis should lead to extensive investigation. When there is clinical suspicion for CS, the patient should undergo one of those novel imaging, non-invasive techniques (either 18F-FDG PET scan or CMR). Positive imaging results in combination with initial findings (step 1) are in favour of CS. Instead of novel imaging techniques, a myocardial biopsy can be taken. If this shows sarcoidosis, then the diagnosis of CS is being made. Because the sensitivity of histology is limited, it would be desirable to avoid this invasive and potentially harmful procedure. Negative histological findings do not rule out CS, and then additional imaging investigations are needed to establish the diagnosis, using either CMR or PET scan.

Step 3: If there are no clinical or laboratory findings indicating cardiac involvement, the patient should be followed-up, by repeating tests (referred in step 1) every 1or 2 years and ultimately whenever new cardiac symptoms arise.

Step 4: After establishment of the diagnosis (i.e. after a positive biopsy, CMR or PET), the patients will be treated with intensive corticosteroids.

**Fig. 1:**
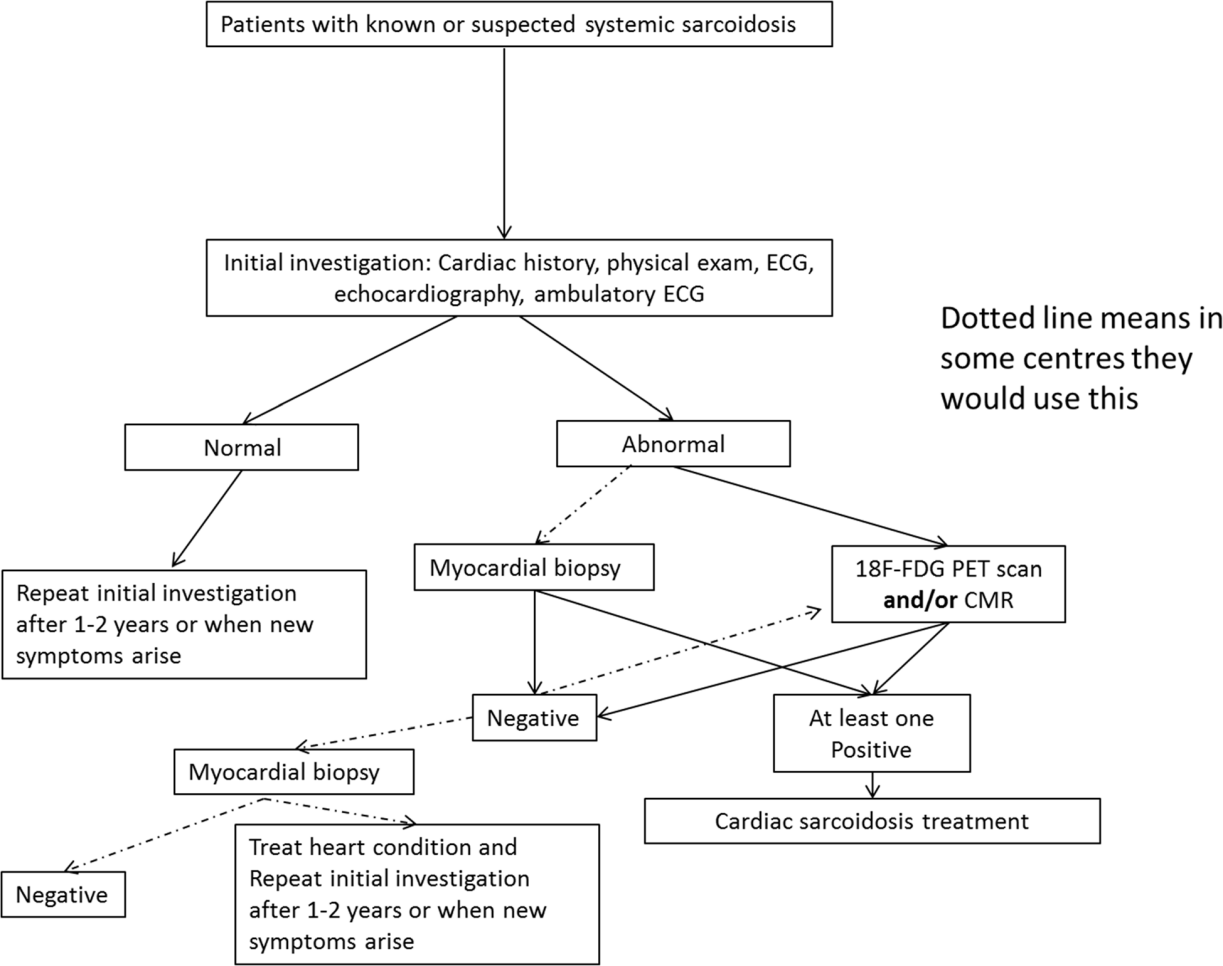
Possible clinical pathway of diagnosis of cardiac sarcoidosis

The following figure summaries a possible clinical pathway (Fig. 1):

### Prior tests and alternatives

Prior tests include 12-lead ECG, ambulatory ECG (24-h or longer ECG Holter), transthoracic echocardiogram (TTE).

No other alternative tests are currently in use.

### Rationale

The limited yield of endomyocardial biopsy, and the limited accuracy of various clinical criteria, outlines in essence the absence of ‘gold standard’ diagnostic criteria. Consequently, CS may be misdiagnosed, with often severe implications for the patient, including avoidable sudden, cardiac death. On the other hand, when diagnosed in good time and appropriate treatment is offered, the survival rate approaches the one of healthy individuals. Hence, timely diagnosis is of paramount importance.

### Objectives

Characterize and compare the diagnostic accuracy of cardiac MRI, FDG-PET and myocardial biopsy for the diagnosis of cardiac sarcoidosis.

## Methods

### Criteria for considering studies for this review

#### Types of studies

We consider diagnostic test accuracy studies (case-control or consecutive series) of any individual index test (CMR, 18F-FDG PET, biopsy—see below) against the reference standard Japan Criteria or equivalent. For case-control studies, we will only use available data from the ‘cases’ arm of the study, since the ‘control’ arm would not fulfil the participant inclusion criteria.

As the revised Japan criteria were published from 2006 onward, we will also consider studies using an exact equivalent of the Japan Criteria, so that we do not omit any papers with relevant evidence. We will perform sensitivity analysis on those studies if a sufficient number of such studies was to be included. We will exclude studies that are reported only in abstract form, are uncontrolled reports (case series, case reports) and are randomized controlled trials of test-treatment design that are more appropriately analysed as intervention than as diagnostic test accuracy studies.

#### Participants and target conditions

Adult patients (aged 18 years or older) with established, pre-existing, sarcoidosis (extra-cardiac) or having established isolated cardiac sarcoidosis

#### Index tests and reference standard

Due to the complex relationship between the de-facto reference standard in clinical practice (the Japan Criteria) and a test sometimes seen as a reference standard, which is however hardly used due to its invasive nature and low sensitivity (myocardial biopsy), for the purpose of this review, the Japan Criteria will be used as a reference standard. Accordingly, biopsy as well as CMR and 18F-FDG PET will be used as index tests. See Statistical Analysis and Data Synthesis section for more details.

### Search methods for identification of studies

Both electronic searches and search of other resources will be performed.

#### Electronic searches

For identifying any eligible studies, we will search the following electronic databases:

- The Cochrane Central Register of Controlled Trials (CENTRAL, The Cochrane Library)
- the Cochrane Register of Diagnostic Test Accuracy Studies
- MEDLINE, Ovid SP (1956 to date)
- EMBASE, Ovid SP (1982 to date)
- ISI Web of Science (1950 to date)
- CINAHL, EBSCO host (1980 to date)

When searching the databases, we will use both subject headings and free text terms. We will develop a MEDLINE search strategy and will adapt it for searching all other databases. We will also search the following regional electronic bibliographic databases, subject-specific databases and dissertation and theses databases:

- IndMed
- KoreaMed
- LILACS
- Panteleimon
- PASCAL
- Google Scholar
- Turning Research into Practice (TRIP) database
- DissOnline
- OpenSIGLE

We will not apply any language restrictions.

A preliminary search strategy for MEDLINE is provided in the Appendix.

#### Searching other resources

For identifying any additional published, unpublished and ongoing studies, we will search the Science Citation Index and check the references of all the relevant studies. We will also search the guidelines by the European, American and Japanese societies in the field.

### Data collection

Pairs of two authors will independently assess the studies for inclusion based on the criteria. Any discrepancies will be resolved by discussion and consensus with a third author external to the assessing pair. We will initially screen studies by the title and abstract and then retrieve full reports for potentially relevant studies. For these studies, we will use a predefined electronic spreadsheet to assess and document studies for inclusion and exclusion according to the above selection criteria. We will also document study selection in a flow chart.

### Data extraction and management

Data extraction will be performed independently and in duplicate using a predefined electronic spreadsheet within the database MS Access. We will resolve disagreements by discussion or by involving a third arbiter. We will then transfer data to RevMan, Stata 14 and to R for further calculations.

### Assessment of methodological quality

Assessment of methodological quality will be performed independently and in duplicate using a predefined electronic spreadsheet. We will resolve disagreements by discussion or by involving a third arbiter. We will use both the Standards for Reporting of Diagnostic Accuracy (STARD) and all four domains from the QUADAS-2 tool [31], a revision of the original QUADAS tool [31], to assess the methodological quality of the included studies. We will also use the public user testing version of the QUADAS-2C for comparison of index tests. This will include the risk of bias with signalling questions and applicability judgement. Both a description and the judgement (coded ‘yes’, ‘no’ or ‘unclear’) for each signalling question will be presented. Additionally, risk of bias and applicability will be coded as ‘high’, ‘low’ or ‘unclear’.

We will pilot the quality checklist independently on a sample of five papers and will refine the checklist before proceeding further, if needed.

When necessary, we will contact the authors of original studies for information on unclear quality items. We will present the items on methodological quality assessments in a methodological quality summary figure. In addition, we will present a methodological quality graph showing the relative distribution of methodological quality assessments for each included study.

### Statistical analysis and data synthesis

For each included study, we will treat the index test results as separate binary classifiers and record details for dichotomization, if appropriate and available. We will collect details on definitions of positive and negative reference standard responses. We will further construct 2 × 2 tables of test and reference standard results to show the cross-classification of reference standard and test outcome. In studies where multiple index tests are performed, we will also construct a series of 2 × 2 tables where the results of investigations will be combined provided that they are derived from the total study population and that the definition of a positive result for combined tests will be reported.

Sensitivity and specificity of each test will be used as the underlying parameter for further calculations. As in clinical practice, health care professionals usually strive to avoid false negatives; sensitivity will be considered the most important property when comparing diagnostic accuracy between tests.

To visualize results, we will provide forest plots showing pairs of sensitivity and specificity together with 95% confidence intervals (95%CI) from each study. We will generally follow a bivariate meta-analysis approach to analyse pairs of sensitivity and specificity using a generalized linear mixed model as outlined by Chu et al. [32].

To deal with the complex relationship of index tests available, we will use a four-part approach to meta-analysis:

- Part 1: Direct comparisons of both CMR and 18F-FDG as index tests to the reference standard Japan Criteria
- Part 2: An indirect comparison of both CMR and 18F-FDG as index tests to the reference standard Japan Criteria
- Part 3: An indirect comparison of both CMR and 18F-FDG with the addition of biopsy as a third index test, again to the reference standard Japan Criteria
- Part 4: A Bayesian meta-analysis using the results of the comparison of biopsy to Japan Criteria as informative prior for both direct and indirect comparisons of CMR and 18F-FDG to the Japan Criteria (analogous to part 1 and 2)

For all parts, only separate index tests, but no combination of index tests (e.g. combined accuracy of performing both CMR and 18F-FDG) will be considered.

For parts 1–3, we will use the ‘lme4’package in R [33] for pooling estimates and especially the ‘glmer’-function for the bivariate binomial method. We will present sensitivity and specificity, logit transformed from the bivariate estimates with 95% confidence intervals. We will provide specificity vs. sensitivity plots showing estimates of individual studies, summary ROC points (summary sensitivity and summary specificity) as well as 95% confidence regions around the SROC characteristic points.

For indirect comparisons (parts 2–3), we will follow the methods generally outlined by Partlett and Takwoingi [34] and recently successfully applied in this context by us [35]. This method allows the indirect comparison of index tests by including a covariate for test type in bivariate models (i.e., meta-regression). For pairwise, between-index test difference comparisons, we will use a bivariate mixed-effects regression model to test the joint null hypothesis of no difference in sensitivity and specificity between two index tests as described above. We will formally compare models using a likelihood ratio test. If the joint null hypothesis is rejected, we will compare sensitivity and specificity individually.

For part 4, we will use the methods described by Menten and Lesaffre [36], especially a network-based hierarchical latent class model. This approach again allows for indirect comparisons of index tests and especially deals with situations where no reliable reference standard is available. We will present prior and posterior distributions of sensitivity and specificity, as well as variance and correlation parameters.

### Investigations of heterogeneity

To explore heterogeneity, patient demographics (for example age, sex, weight) and clinical setting (in-hospital vs. out-hospital), as well as differences in the population (pre-established vs. suggested CS), will be considered potential covariates.

### Sensitivity analyses

We will follow a standard approach by assessing the impact of excluding studies based on QUADAS-2 domains [37], as well as explicitly excluding case-control studies, if there is a relevant number of such studies. We will also assess the impact of a different version of the reference standard used, including versions modified not to include any of the index tests, by performing separate analysis for each version.

We will also compare different modelling approaches as outlined above as parts 3 and 4.

### Assessment of reporting bias

Because there are no commonly accepted methods for assessing reporting bias in diagnostic test studies and such testing might even be misleading in this context, we will not perform such analyses [38].

## Discussion

The most widely recognized diagnostic algorithm for cardiac sarcoidosis is considered out of date, as it precedes the introduction of imaging techniques in diagnostic pathways. These novel imaging techniques, like CMR and FDG-PET scan, have emerged as promising diagnostic tools which may fill the current diagnostic gap. Thus, a systematic review and evaluation of CS diagnosis are much needed. Such an attempt is anticipated to alter the current diagnostic guidelines for CS by shedding more light on the role of sophisticated imaging techniques on prompt CS therapy and follow-up.

## Data Availability

Not applicable (protocol only).

## Appendix

Search strategy for MEDLINE via Ovid.

Epub Ahead of Print, In-Process & Other Non-Indexed Citations, Ovid MEDLINE(R) Daily and Ovid MEDLINE(R) <1946 to Present>

1. exp sarcoidosis/ or (sarcoidosis or Sarcoidoses or (Boeck* adj2 (Disease or sarcoid)) or (Schaumann* adj Syndrome*)).ti,ab,kf.
2. (cardiac* or cardio*).ti,ab,kf,hw.
3. 1 and 2
4. exp MAGNETIC RESONANCE IMAGING/
5. (MRi or NMRi or CMR).ti,ab,kf.
6. ((magn*or MR or MTC or MT or NMR or spin or chemical shift or diffus*) adj3 (imag* or tomogra$)).ti,ab,kf.
7. Diffusion-weighted.ti,ab,kf.
8. or/4-7
9. exp POSITRON-EMISSION TOMOGRAPHY/
10. ((pet adj3 scan*) or (positr* adj4 tomogr*) or pet-ct or ‘pet/ct’ or petct or fdg-pet).ti,ab,kf.
11. 9 or 10
12. 8 or 11
13. 3 and 12
14. exp Sarcoidosis/ra, ri
15. 2 and 14
16. 13 or 15

## Abbreviations

18-FDG-PET/FDG-PET/18F-FDG: 18F-fluoro-2-deoxy-d-glucose-positron emission tomography
CENTRAL: Cochrane Central Register of Controlled Trials
CMR: Cardiac magnetic resonance
CS: Cardiac sarcoidosis
LGE: Late gadolinium enhancement
MRI: Magnetic resonance imaging
QUADAS: Quality assessment of diagnostic accuracy studies
STARD: Standards for Reporting of Diagnostic Accuracy
TRIP: Turning Research into Practice
